# Supporting mental health self-care discovery through a chatbot

**DOI:** 10.3389/fdgth.2023.1034724

**Published:** 2023-03-07

**Authors:** Joonas Moilanen, Niels van Berkel, Aku Visuri, Ujwal Gadiraju, Willem van der Maden, Simo Hosio

**Affiliations:** ^1^ Faculty of Information Technology and Electrical Engineering, Center for Ubiquitous Computing, University of Oulu, Oulu, Finland; ^2^ Department of Computer Science, Human-Centered Computing, Aalborg University, Aalborg, Denmark; ^3^ Faculty of Electrical Engineering, Mathematics and Computer Science, Web Information Systems, Delft University of Technology, Delft, Netherlands; ^4^ Faculty of Industrial Design Engineering, Delft University of Technology, Delft, Netherlands

**Keywords:** conversational agent (CA), chatbot, conversational user interface (CUI), mental health, self-care, human-computer trust, human-computer interaction

## Abstract

Good mental health is imperative for one’s wellbeing. While clinical mental disorder treatments exist, self-care is an essential aspect of mental health. This paper explores the use and perceived trust of conversational agents, chatbots, in the context of crowdsourced self-care through a between-subjects study (*N* = 80). One group used a standalone system with a conventional web interface to discover self-care methods. The other group used the same system wrapped in a chatbot interface, facilitating utterances and turn-taking between the user and a chatbot. We identify the security and integrity of the systems as critical factors that affect users’ trust. The chatbot interface scored lower on both these factors, and we contemplate the potential underlying reasons for this. We complement the quantitative data with qualitative analysis and synthesize our findings to identify suggestions for using chatbots in mental health contexts.

## 1. Introduction

Good mental health is imperative for one’s general wellbeing. Conversely, mental disorders cause tremendous social (1) and economic (2) burdens worldwide. Higher education students are especially vulnerable, as they are typically at the peak onset of many mental disorders, such as depression and anxiety (3). However, a staggering number of students suffering from symptoms never seek help, and many seek help far too late in the process (4). To this end, support from one’s community has been identified as a valuable avenue to explore as a complementary mechanism to traditional healthcare and clinical interventions (5). However, knowledge is often sparsely shared within the community due to stigma (6). Novel research approaches and support mechanisms with a lower barrier for participation are required to address this. In addition to helping people with existing mental health conditions, it is important to maintain healthy mental wellbeing for those not feeling particularly ill. Support mechanisms have proved effective for preventive approaches as well (7).

One approach currently investigated for mental health is *self-care*. Self-care, in general, refers to how people take care of their wellbeing or a mental health condition on their own, either using the information found online or as instructed by their caretakers (8). A community sharing a similar burden can be an excellent resource for self-care methods. While various other means of serving these methods exist, researchers are currently actively looking into the affordances of chat-based conversational agents, chatbots, due to their inherent relatability and rapidly increasing interaction capabilities (see, e.g., (9,10)).

**Figure 1:**
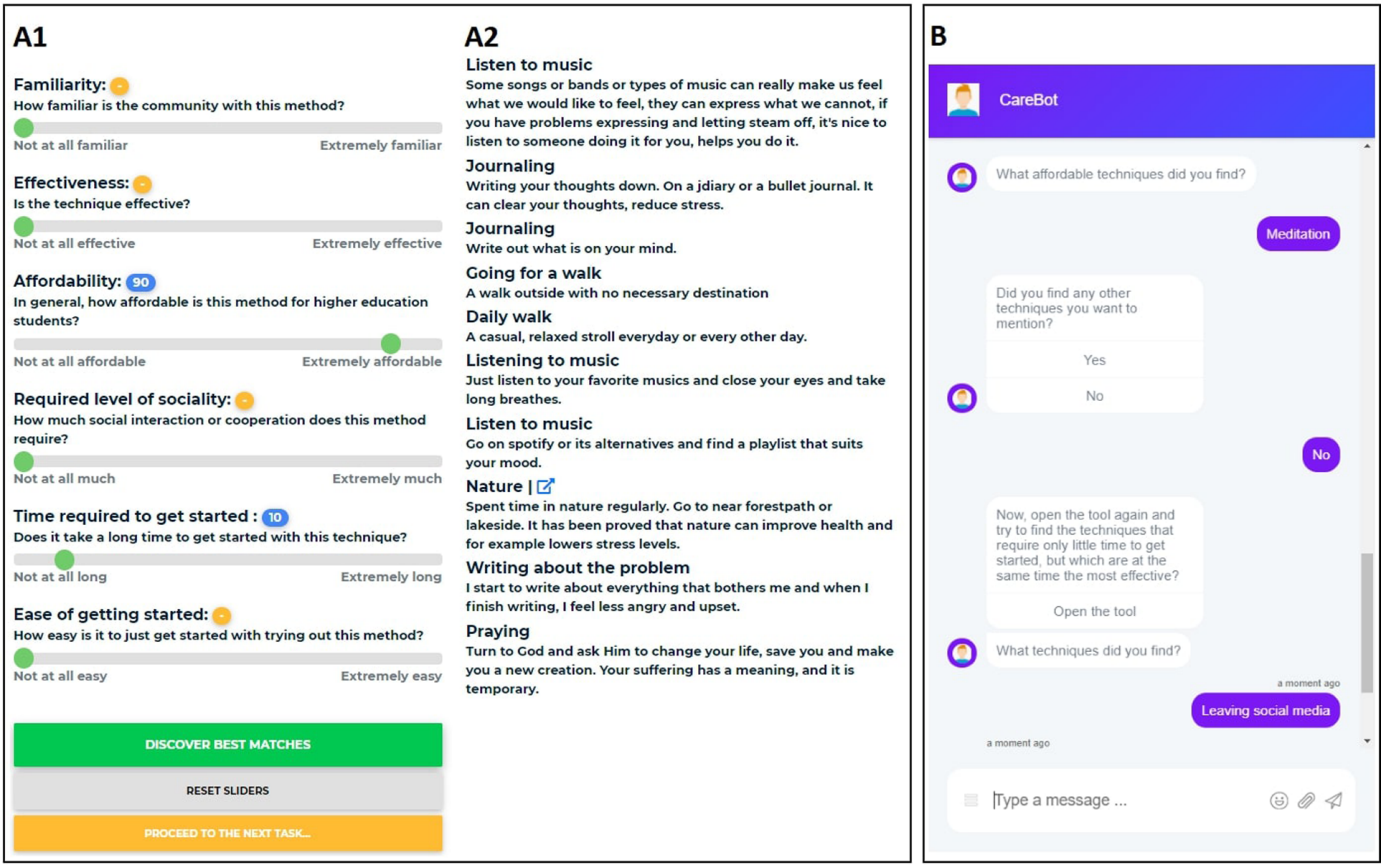
Snippets of our two platforms used in the study. (**A**) The system for mental health self-care discovery with sliders for different criteria (**A1**) and recommended methods (**A2**), based on the slider selections. (**B**) The chatbot with examples of asking found methods and presenting the user with a new task.

In our earlier work (unpublished in academic venues), we have crowdsourced an extensive list of self-care methods among the higher education community to uncover how students maintain and improve their mental health. These methods include, for example, meditation, spending time with others, volunteering, and working out at a gym, with additional methods presented in Figure 1. The students have also cross-evaluated each other’s contributions across a set of specific criteria. In this paper, we used this data to bootstrap a decision support system (DSS) that allows for discovering suitable self-care methods through an online user interface (UI) and by using the same criteria that were used to bootstrap the DSS (see Figure 1). To explore the potential use of chatbots in serving the DSS and trust in the system, we offered the DSS UI to 80 higher education students in a between-subjects study. The study groups consist of two groups of 40 participants through A) a standalone online DSS, and B) the DSS embedded in a narrative served by a conversational interface (see Figure 1).

In this work, we set out to find factors which affect the formed trust between a mental health chatbot and the user. We offer the users hundreds of crowdsourced methods for mental health self-care in an interface embedded in a chatbot conversation and compare that to a traditional web interface. We hypothesize that providing the users with clear instructions and interactive conversation alongside the method discovery could lead to improved trust towards the system.

Our findings highlight that the participants interacting with the chatbot report lower perceived system security and integrity but no significant difference in the overall trust between the DSS group. The human-like behaviour of the chatbot also appears to affect trust for individual participants. Based on our findings, we argue that improving the (perceived) security and integrity of the chatbot will help design more effective chatbots for mental health. Furthermore, we find that using a chatbot for mental health self-care method discovery shows promise, with several participants stating their fondness towards the chatbot. While research in mental health chatbots and their trust is plentiful, we provide contributions to direct comparisons of two systems and how to further improve the trust towards them. In addition, we provide information on how viable these kind of crowdsourced methods are in digital healthcare.

### 1.1. Related work

Chatbots mimic human conversation using voice recognition, natural language processing, and artificial intelligence. Initial versions of chatbots operated purely through text-based communication, aiming to provide intelligent and human-like replies to its users (11). Over the past decade, chatbots have grown in popularity, and together with voice-activated conversational agents such as Apple’s Siri, they have become part of everyday life (11).

#### 1.1.1. Chatbots in mental health

Mental health has been defined by the World Health Organization (WHO) as “*a state of wellbeing in which the individual realizes his or her abilities, can cope with the everyday stresses of life, can work productively and fruitfully, and can make a contribution to his or her community*” (12). Mental health is a suitable context for chatbots due to their ability to provide dynamic interaction without relying on a professional’s availability (13), and the potential for chatbots to provide empathic responses (14).

In this article, we specifically focus on self-care for mental health. Self-care is used both to manage long-term conditions and to prevent future illnesses and has been identified as a critical approach to supporting independence, providing control to the patient rather than solely relying on a clinician, and reducing reliance on an overburdened healthcare system (15). The application of chatbots as self-care tools is a relatively under-explored opportunity, with many open questions regarding identifying, monitoring, and evaluating self-care methods. Here, we focus on using a chatbot as a tool for *discovering* self-care solutions in mental health.

Using chatbots in mental health care has grown in popularity in recent years (16,17) and point to the opportunity to support users in long-term self-care development and effectively communicate goals in response to prior and new user needs. While chatbots are not suitable to provide the users with actual clinical intervention, they are an excellent way to provide mental health counselling, such as presenting the users with various self-care methods to help them improve their mental health (18). While most research for chatbots offering self-care focuses on young people, it has been shown to be effective for older adults, as well, as is shown by Morrow et al., who present a framework for the design of chatbots on health-related self-care for older adults (9). As is found in the review by Abd-Alrazaq et. al. (17), using chatbots for these kind of purposes can improve their mental health, but should commonly be used as an adjunct to intervention with a healthcare professional. In addition, using chatbots in mental health is not without its risks. One of the most crucial things to be taken into consideration when designing a mental health chatbot is its ability to reply accordingly to the user’s messages; for example, poorly managing the responses to suicidal behaviour might lead to serious consequences (16).

Various factors affect the overall effectiveness of a chatbot, but in this research we focus specifically on the user’s trust towards the chatbot. A vital prerequisite to offering mental health through chatbots is to build a level of trust between the user and the chatbot. Müller et al. find that a lack of trust in chatbots results in reduced uptake of these digital solutions (19). Furthermore, there are several factors to be taken into account to further enhance the perceived trust for chatbots, most notably, the chatbot’s personality, knowledge and cognition have shown to increase trust (20). Previous research shows promise in building trust between a chatbot offering counselling and the user (21) and that the use of conversational interfaces as compared to a conventional web interface can lead to better performance and user experience (22). Recent work by Gupta et al. shows a similar setting to ours and an increase to trust when using a chatbot as opposed to a traditional web interface for housing recommendations (23).

#### 1.1.2. Trust in computer systems

Trust and its formation have been important topics in automated (24,25) and online systems (26,27), as well as specifically in chatbots (28). The definition of trust varies, but for this paper, we define it as “*the attitude that an agent will help achieve an individual’s goals in a situation characterized by uncertainty and vulnerability*” (25). An agent can refer to a human individual but also a chatbot. Zhang & Zhang highlight the many factors that affect trust, stating that trusting behaviour is formed from an individual’s trust beliefs, attitude towards trust, and trust intention, which are further influenced by, for example, external environmental factors (26). Work by Tolmeijer et al. shows that trust develops slowly, with the user’s initial trust impression having a large anchoring effect (29).

To measure trust, we used the “trust in the automation” scale by Jian et al. (24), which is one of the most widely used scales for measuring trust. Several other scales for measuring trust exist, but as most revolve around the same core topics and the scale by Jian offers easily interpretable results, we deemed this scale suitable for our purposes. Extensive research by Nordheim (30) shows that trust in chatbots is formed with factors such as risk, brand, and expertise, which are covered in our survey questions, presented in Table 1. In addition, the concept of trust seems to be similar for both human-human and human-machine situations (24).

**Table 1:** Questions of the ‘trust in automation’ scale used to measure trust.

#	Question
Q1	The system is deceptive
Q2	The system behaves in an underhanded manner
Q3	I am suspicious of the system’s intent, action, or outputs
Q4	I am wary of the system
Q5	The system’s actions will have a harmful or injurious outcome
Q6	I am confident in the system
Q7	The system provides security
Q8	The system has integrity
Q9	The system is dependable
Q10	The system is reliable
Q11	I can trust the system
Q12	I am familiar with the system

All items use a 7-point Likert scale. These questions are derived from the work by Jian et. al. (24).

## 2. Materials and methods

### 2.1. Apparatus

#### 2.1.1. Crowdsourcing decision support system

We used a publicly available lightweight crowdsourcing tool developed by Hosio et al. (31) to collect and assess mental health self-care methods. The tool is implemented using HTML, Javascript, PHP, and MySQL, and can be deployed on any website using a standard HTML iFrame tag. We will refer to this tool as the Decision Support System or **DSS**. The DSS has three main components; users can search for methods, rank existing methods, and input new methods to the system. A similar system framework has been adapted to other studies, e.g., for crowdsourcing treatments for low back pain (32) and personalized weight-loss diets (33).

In the context of this work, the study participants use the search component. Using the decision support interface, as depicted in Figure 1A, participants can search for self-care methods through a configuration of six different sliders that adjust familiarity, effectiveness, affordability, required level of sociality, the time required to get started, and ease of getting started. After the sliders have been adjusted, the tool presents the participant with the mental health self-care methods that best match the criteria configuration. Each of the six characteristics was rated by the users of the tool during the data collection.

Data collection of the shown methods was conducted before this study. The tool was made publicly available, and it was used to collect new mental self-care methods from its users and requested users to rate and validate pre-existing methods in the system. Methods were collected from over 900 participants, and over 30 000 individual ratings for hundreds of different self-care methods were obtained during the study. In addition to these methods, the participants were asked for open feedback on where, how, and why they seek self-care-related information.

As these components of the tool are not the focus of this article, we point the reader to (32) for more information about its functionalities.

#### 2.1.2. Chatbot implementation

In this study, we were interested in exploring whether wrapping the tool in a conversational interface where participants could converse with an agent would affect the perceived trust or other aspects of the system. We purchased a license to BotStar^1^ to use as the chatbot. BotStar supports opening external URLs in a full-screen modal popup as part of the conversation flow, which is how we embedded the DSS among the scripted conversation. A snippet of the used conversation script can be seen in Figure 1B. The chatbot was fully implemented via BotStar and was launched on a remote WordPress page.

#### 2.1.3. Post-task survey

After completing the three tasks of using the DSS either through the web interface and instructions, or while interacting with the chatbot, each participant responds to a final questionnaire through Google Forms. The final questionnaire contains the trust in automation scale items (see Table 1) using a 7-point Likert Scale (1 = Strongly Disagree, 2 = Disagree, 3 = Somewhat Disagree, 4 = Neutral, 5 = Somewhat Agree, 6 = Agree, 7 = Strongly Agree) and three open-ended follow-up questions;

• **F1**: We asked you to search for mental health self-care methods with criteria of your own choice. What are your thoughts on the results?
• **F2**: What kind of support do you expect from a system offering mental health self-care techniques?
• **F3**: What features affected your trust in the system?

#### 2.1.4. System overview

**Figure 2:**
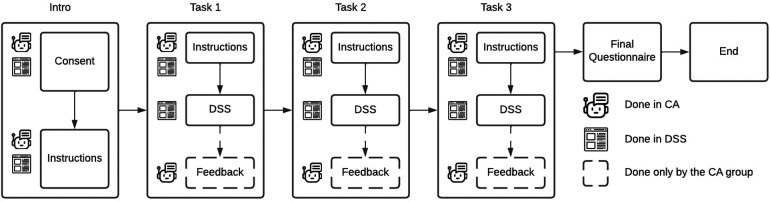
Flow diagram of the study design for the two study groups. The WEB group does every part of the study in the DSS, while the CB group switches from the chatbot to embedded DSS only for task completion. The chatbot briefly asks, what methods the user found from the CB group.

The full study system setup consists of the following components, and is presented in detail in Figure 2:

• **Prolific**: The study is deployed on the Prolific^2^ crowdsourcing platform, where participants are given instructions and links to proceed to their study tasks.
• **DSS Platform**: The decision support system is deployed as a web interface on our remote server.
• **Chatbot**: The chatbot is self-hosted using BotStar on our remote server. For the chatbot participant group, the DSS platform is opened inside the chatbot using embedded web views.
• **Final Questionnaire**: The final questionnaire for the participants is deployed using Google Forms.

### 2.2. Experimental setup and protocol

For this article, the two study groups are named and referred to as follows:

• **CB group**: Group using the chatbot that wraps the online DSS
• **WEB group**: Group using only the online DSS.

Participants were recruited from Prolific, an online crowdsourcing platform. The participants were pre-filtered to higher education students using the platform’s quality control mechanisms. Participants were rewarded USD2.03–USD2.54 based on a task duration of 11–15 min. Participants are anonymous; thus, no approval for human subject research was needed beyond our project-wide approval from our University’s ethics board. Participants were asked to give consent at the beginning of the study and could terminate their participation at any point of the study.

We asked participants from both groups to look for self-care methods three times; first to explore methods that are the most affordable, then methods requiring only a little time to get started but which are at the same time the most effective, and finally methods that would suit the participants’ own needs the best. In the CB group, these instructions were given by the chatbot, and in the WEB group, the instructions were given on top of the web page in a simple notice box.

In the CB group, we focused on making the narrative realistic and neutral tone. To this end, the chatbot walks the participants through the process of discovering self-care methods, providing them with detailed instructions on what to do next (Figure 1B). To add a level of human-like conversation between the chatbot and the participant, the participant can be called by a nickname. The chatbot greets them at the beginning and expresses their gratitude at the end of the session. In the middle of the conversation, the participants are prompted to complete the same tasks as in the other experimental condition. At the end of each task, the chatbot asks what methods the participant found during this round. To ensure comparability between conditions, participants use the same tool for identifying a suitable self-care method.

After completing the three aforementioned tasks of identifying self-care methods, participants were directed to the final questionnaire. To evaluate the trust and credibility in both conditions, we use the 12-item questionnaire for trust between people and automation proposed by Jian et al. (24) presented in Table 1. This scale has been created using large amounts of empirical data and helps us understand how different system characteristics affect users’ trust. In addition, we ask three open-ended questions to determine what the users think of the methods recommended for them, what kind of support they expect from a system offering mental health self-care methods, and which factors affected their trust in the system. This study setup is presented in Figure 2

## 3. Results

### 3.1. Participant demographics

43 of the participants identify themselves as male and 37 female. The average age was 23.26 (SD = 4.59) years. 72 of the participants reside in Europe, the two most represented countries being Portugal (*N* = 20) and the UK (*N* = 13). 52 participants were undergraduate students, 24 graduate students, and 5 doctoral students. The mean age for the two groups was 24.1 (SD = 5.89) years for the CB group versus 22.43 (SD = 2.44) for the WEB group.

### 3.2. Quantitative analysis

**Figure 3:**
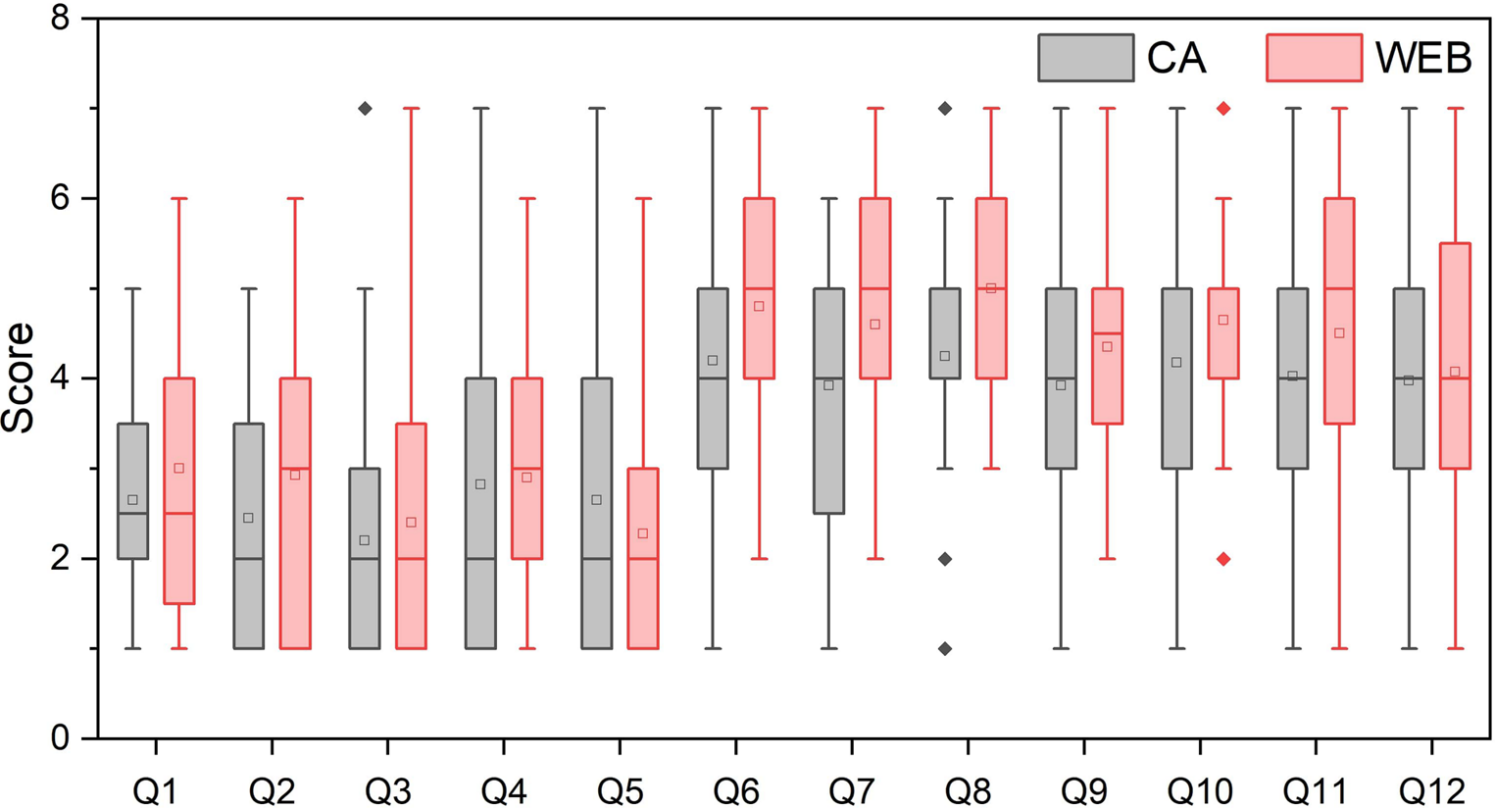
Scores of the trust in automation scale items for both study groups. Student’s t-test shows significant difference for Q7 (*The system provides security*): CB: 3.93 (SD = 1.58), WEB: 4.6 (SD = 1.34), *t* = −2.07, *p* = 0.042, Cohen’s d = 0.46, and Q8 (*The system has integrity*): CB: 4.25 (SD = 1.46), WEB: 5 (SD = 1.18), *t* = −2.53, *p* = 0.014, Cohen’s d = 0.57. As the performed t-tests were independent of each other, there was no need for Bonferroni corrections.

Scores for the trust in automation scale are presented in Figure 3. The CB group reports approximately half a point lower values for both the security and integrity question than the WEB group with a significant difference (*p* < 0.05). For the other questions, the difference between the two groups is similar, but with *p* > 0.05, so these findings are not statistically significant. In addition, we found a significant difference in positive trust, with the CB group reporting approximately 0.5 lower scores compared to the WEB group (*p* < 0.05).

#### 3.2.1. Trust scores

Trust scores for the *trust in automation* survey were determined by taking the combined means of the positively worded items (*Q6-Q12*) and reverse-scored negatively worded items (*Q1-Q5*). Similarly, trust scores for positive and negative items were determined. For the overall and positive trust scores, a higher value indicates a bigger trust, whereas, for the negative trust scores, a lower value signifies a bigger trust (24).

**Positive trust:** CB: 4.07 (SD = 1.25), WEB: 4.57 (SD = 0.95). *t* = −1.98, *p* = 0.03, *Cohen’s d*: 0.45.

**Negative trust:** CB: 2.56 (SD = 1.20), WEB: 2.7 (SD = 1.33). *t* = −0.51, *p* = 0.31, *Cohen’s d*: 0.11.

**Overall trust:** CB: 4.64 (SD = 1.12), WEB: 4.87 (SD = 0.98). *t* = −0.96, *p* = 0.17, *Cohen’s d*: 0.22.

### 3.3. Qualitative analysis

To obtain a more in-depth understanding of the underlying reasons behind participants’ differences in trust, we conducted a thematic analysis of the participants’ feedback responses F1–F3 as collected directly after using the system. This analysis was conducted by using conventional content analysis (34) in which two of the paper’s authors tagged the feedback responses in a shared online document with key themes present in the given response. In case of disagreement in categorizing participant responses, a third author was included in the discussion. We present the three themes that we identified to affect participant trust; “personal experiences,” “perceived reliability,” and “presentation of results.” These themes are present in both two study groups and we found no significant difference to how often each theme appeared between the CB and WEB groups.

#### 3.3.1. Personal experiences

The ability to relate presented results to one’s own experiences strongly affected participants’ trust. Being presented with familiar results leads to participants being more receptive to methods they had not seen before. One participant discusses this notion in terms of establishing initial trust in the system; “*I found that my trust was established when I saw many techniques that I use, like mindfulness, running, etc. I think it was how familiar I was with the techniques shown that established my trust.*” (P21). On the other hand, one participant that had poor experiences with some of the methods suggested by the system expressed that the inclusion of these methods negatively affected their trust level; “*disagreeing with some of the suggestions made me question how good it was*” (P25).

In addition to relying on their own experience in assessing and evaluating presented methods, several participants also expressed their wish for a future system to incorporate their own experiences to provide more accurate suggestions. “*I expect the system to be able to assist me in choosing the most suitable techniques for me, giving me the right information and listening to my needs*” (P14). Participants’ responses indicate that they are willing to put in the additional effort required to provide this data if it would result in more valuable suggestions; “*A short questionnaire done previously so that the system can make more accurate suggestions.*” (P05). Such an approach may also help present the mental health self-care methods a participant already has experienced differently.

#### 3.3.2. Perceived reliability

While our quantitative results indicate that participants typically trusted the results presented by the system, our analysis of the open-text responses also highlights why some participants indicated a lower level of trust in the presented results. A couple of participants highlighted a lack of information on the people who contributed to the system’s data as an obstacle to building trust. “*Not knowing much about the ‘community’ behind the system.*” (P26) and “*You cannot know precisely who is suggesting what.*” (P47) highlight these perspectives.

In line with the aforementioned theme of personal experiences, our participant responses indicate a tight balance between novel suggestions to which participants might express some uneasiness and well-known, established ideas that participants could dismiss as too obvious. For example, one participant highlighted that they had high trust in the presented results but that the methods were not extremely helpful to them; “*I thought the techniques would show me something ‘new’ that I have not heard of yet, but I have already tried most of them. I also think the techniques are too focused on depression and anxiety, which I guess is what most people suffer from, but I was expecting something more…groundbreaking?*” (P75). On the other hand, other participants commented that the lack of novelty did not affect their perceived usefulness or trust in the system; “*I think the results were rather expected, but that does not make them worse in any way. The system provided simple solutions and great ideas overall.* (P27), indicating that being familiar with the suggestions increases the perceived reliability of the overall system. Even as participants may have experience with some of the presented self-care methods, this did not necessarily deter them from trying out any of the other methods; “*Some of the techniques do not work for me, but some I have not thought about it and might be good to give them a try.*” (P31).

#### 3.3.3. Presentation of results

Lastly, a large part of the participant sample showed how results were presented as a trust-affecting factor. Several participants mentioned a lack of uniformity in presenting self-care methods as having a negative effect. While this lack of uniformity directly results from the crowdsourced nature of the self-care methods and our deliberate choice not to edit participant contributions, some of these issues can be addressed relatively quickly in future iterations. For example, one participant commented on the capitalization of the methods “*The results […] were not written uniformly (some lacked capitalization, other did not, which is fine but weird)*” (P10). Similarly, another participant highlighted grammatical errors, as well as inconsistency in capitalization, as a trust-impeding factor in “*poor capitalization/non-standard grammar*” (P18). Although participants identified these errors as problematic, this lack of ‘strict’ grammar and spelling also highlighted to the participants that these methods were contributed by other users; “*it seems that a lot of the options were submitted by users, the spelling was wrong in some places too*” (P34)

Without explicitly being asked to do so, several participants commented positively on how the chatbot presented the system to the user; “*The fact that the system used a very humane, calm and warm language. The fact that it called me by nickname also affected my trust in the system.*” (P14). Similarly, another participant mentioned that their trust was positively affected by “*[…] the aesthetic and the reassuring phrases*” (P42). While not made explicit by the participants, the sensitive nature of mental health can play a significant role in this expressed sentiment.

While participants typically found the presented discovery interface useful, they also highlighted that additional information would be helpful. In particular, a couple of participants highlighted that the tool could do more to help them on their way once a method had been selected; “*Other than listing the options, as the tool had greatly done, I would also love a description on how to get started with each technique.*” (P21).

## 4. Discussion

### 4.1. Using chatbots in a self-care discovery system

Chatbots are forecast to ease the looming resource crisis in healthcare and automate many customer service functions in general. Mental health is a high-impact and sensitive domain. Thus, any interactions must be safe, secure, and confidential. To this end, crowdsourcing systems can complement ‘official’ clinical care: a repository of user-contributed self-care methods is simply another way to structure people’s ideas and content, much like an online forum. However, before deploying this kind of crowdsourced system into real use, significant work has to be done to ensure it works as intended. In our research we did not receive any major feedback on potentially harmful or undesired methods, due to researches having gone through the list of methods beforehand. To take this into account in similar systems, we believe, that a moderator or automated recognition could increase the system’s safety to be viable in mental healthcare.

Our study compared a standalone decision support interface to a setup wrapped in a conversation with an artificial agent. We found that the version wrapped within a conversation with a chatbot led to a lower trust for the system’s security and integrity. While the exact reasons for this cannot directly be identified, we speculate that one explanation might be the privacy concerns that are frequent among online mental health services (20, 35). Participants could not be identified from the conversations with the chatbot, but we asked the users whether they would like to be called by a nickname to make the chatbot more humane and empathetic and act like most chatbots online do. Participants might have felt this makes them more identifiable and interferes with their privacy needs. This might also be due to the study design, as the nickname was asked only from the CB group. Other possible explanations for the lower scores could be the preference to use such a system without additional guidance and the general uncertainty towards the chatbot. Subsequent studies, perhaps using a within-subjects design, could help determine why the chatbot seemed to degrade these scores.

This study gives a clear direction to create a broadly accessible system for helping people to maintain and improve their mental health. With an active user base, new self-care methods could be introduced and ranked as they continuously interact with the system. Integrating a self-care discovery system within the conversation can make it more approachable and easily accessible (36). We believe that interaction with the chatbot can also improve its overall usability and performance, thus potentially increasing the effect it can have on the user’s mental health. To achieve this, the trust towards the system using the chatbot needs to be improved.

### 4.2. Transparency, integrity, and security

One of the other factors affecting chatbot trust is transparency, i.e., sharing the limitations of the chatbot with the users to help them predict different outcomes and conversations, with other affecting factors including dialogue, interface, expressions, and conversational styles (28, 37). Here, transparency was also clearly an issue with the content itself: Users wanted more details about who articulated, assessed, and helped build the knowledge base of self-care methods. As our qualitative analysis shows, this negatively affected the trust for both of the two groups. Much uncertainty comes from the recommended self-care methods and how those have been added to our system.

Second, the difference between the integrity and security scores might also come from the fact that the CB group uses two different systems instead of one for the WEB group. Participants’ experienced that if the service they are using is fragmented to multiple interfaces, this could potentially create more points for attack for privacy intrusions. When the number of used systems, applications, websites, and similar increases, the risk of being exposed to data breaches increases. Some might feel uncomfortable sharing private information if it is required to share it with multiple sources. Thus, we believe that implementing this method discovery directly in the conversation could lead into better trust, without the need to do that in a separate system.

### 4.3. Chatbot behaviour and humanity

Chatbot behaviour and human likeness are essential factors in forming trust in chatbots (9, 20, 38). There is evidence that the personality of the user makes a difference in how trust between the user and the chatbot is formed (19, 37). In line with our findings, previous studies have frequently mentioned human likeness as a key aspect of trust (9, 38). Indeed, in our study, six participants directly mentioned how the chatbot and its human-likeness positively affected their trust in the system. Some contradicting evidence emerged in a study by Folstad et al. (38) on chatbots in customer service, where the majority of participants preferred human-likeness and personalized chatbots for building trust. However, some participants referred to the uncanny valley effect. To conclude, it is essential to keep a chatbot identifiable as non-human when developing trustworthy systems. Our chatbot was designed to be identifiable as non-human from the beginning with qualities such as its introduction, speech patterns, and name, *CareBot*. A

We believe our results might have been significantly different with a different kind of bot personality, but having the chatbot play as neutral of a role as possible could yield the best results in the mental health context. Naturally, chatbots with varying personalities are excellent avenues to explore in future work.

### 4.4. Chatbot as a factor affecting trust

Although some participants did mention that the chatbot affected their trust in the systems, the amount was lower than expected. Most participants focused on describing the factors affecting their trust in the self-care discovery tool while disregarding the chatbot. While we made sure to keep the connections between the two systems seamless by, for example, implementing the self-care discovery system within the chatbot window instead of a separate web-browser tab, this is something to focus on more in the future. An excellent way to handle this would be to have the chatbot ask for the criteria and give the recommended methods to the user directly, without needing a tool of its own. This could make the system easier to use while also giving more possibilities to explain the methods and their practical usage to the participants, one feature requested by the users and discussed in the *presentation of the results* section. Transparency should remain, and the sources of the recommendations offered should always be explained and referred to.

As it stands, the created chatbot might not yet be trustworthy enough for self-care method discovery, as the standalone DSS version enjoys larger overall trust. While we got significant results only for the security and integrity between the two conditions, those are crucial factors for a successful chatbot and might lead to users not being comfortable using the chatbot due to, for example, security concerns. Neglecting these factors can lead to significantly lower trust compared to standalone systems. This gives us a clear direction for improving the chatbot.

### 4.5. Limitations and future research

To gain a more in-depth understanding of how users form trust in the specific DSS used here, a larger sample could be beneficial. We also compared results only between two groups; one group using the WEB interface wrapped in conversation and another group using only the WEB interface but were missing a study condition where the full interaction is conducted through the chatbot. The DSS interface was included in both groups. Even though the DSS interface was presented to the CB group embedded in the chatbot interface, it remains a web interface at its core. In future studies, it would be important to see how a conversational agent performs without the need of external interfaces outside of the chatbot. For this study, due to technical limitations, this feature was not yet implemented.

The original research by Jian et al. (24) shows, that trust, and distrust (positive and negative trust) have a negative correlation, and thus there is no need to develop separate scales for the two conditions. The scale used is their proposed way to measure overall trust, but their findings also suggest that calculating the positive and negative trust scores might be unnecessary. We are also aware of other existing measurements and standardized surveys for trust. In our work we decided to use a widely accepted and used survey to gain first insights to how trust might change when using a conversational agent for mental health self-care intervention. However, in future research, it might be beneficial to use surveys made to measure trust in a health or web-based context instead of a more generalized one (39,40).

As mentioned before, previous research shows that the human-likeness of the chatbot might significantly affect the formed trust between them and the user. This could be especially important in mental health applications. To best compare the use of chatbots within self-care discovery systems, chatbots with differing personalities and levels of anthropomorphism could be used.

## 5. Conclusion

We presented an exploration of using a chatbot for self-care method discovery, specifically focusing on perceived trust in the system. The use of a chatbot was compared to a traditional standalone web interface. We found significant differences between security, integrity, and the positive trust between the two conditions, and that trust is affected mainly by personal experiences, perceived reliability, and presentation of results. To improve the trust for the chatbot further, more attention is needed for its security and integrity, which could be done by, for example, implementing the self-care method discovery directly within the conversation.

Although the results for the trust survey showed lower trust in all categories for the CB group, several students mentioned the chatbot to positively affect their trust in the system. We believe improvements to the chatbot, especially to increase its security and integrity, could indeed increase the trust to the same level as the WEB group. Using a chatbot for self-care method discovery could make this kind of system more easily accessible, easier to use, and overall increase the user experience if the overall trust towards it is high enough.

## Data Availability

The datasets presented in this study can be found at https://doi.org/10.6084/m9.figshare.22193803.v1.

## Footnotes

^1^ https://botstar.com/

^2^ https://www.prolific.co/
